# The impact of music intervention during emergency suturing on patients’ pain and anxiety: a meta-analysis

**DOI:** 10.3389/fpubh.2026.1713057

**Published:** 2026-01-27

**Authors:** Xiaotian Zhou, Zehua Li, Tianshu Mei, Meng Fang, Ping Huang

**Affiliations:** 1 Department of Emergency, Nanjing Drum Tower Hospital, Affiliated Hospital of Medical School, Nanjing University, Nanjing, Jiangsu, China; 2 School of Nursing, Nanjing University of Chinese Medicine, Nanjing, Jiangsu, China

**Keywords:** anxiety reduction, emergency suturing, meta-analysis, music intervention, pain management

## Abstract

**Objective:**

To evaluate the effects of music intervention on pain and anxiety in patients undergoing emergency suturing.

**Methods:**

PubMed, Embase, Web of Science, and the Cochrane Library were systematically searched up to September 9, 2025. Randomized controlled trials comparing music intervention with standard care during emergency laceration repair were included. Standardized mean differences (SMDs) with 95% confidence intervals (CIs) were pooled using a random-effects model. Heterogeneity was assessed using the I^2^ statistic, and evidence certainty was evaluated with the GRADE approach.

**Results:**

Four RCTs involving 388 patients were included. Music intervention significantly reduced pain compared with standard care (SMD = −0.28, 95% CI − 0.48 to −0.08; I^2^ = 0%). Anxiety scores showed a borderline reduction in the music group (SMD = −0.40, 95% CI − 0.80 to 0.00; I^2^ = 20%). Sensitivity analyses supported the robustness of the results. The certainty of evidence was rated as low to moderate.

**Conclusion:**

Music intervention during emergency suturing significantly reduces pain and may provide modest benefits in alleviating anxiety.

## 1 Introduction

Pain and anxiety are among the most frequent complaints of patients receiving emergency wound care, especially during suturing. These procedures are commonly linked to physical discomfort, psychological stress, and fear, which adversely affect patient experience and procedural cooperation (1, 2). Although local anesthetics and sedatives are routinely used, they may not completely relieve patients’ distress and can introduce additional risks, especially in vulnerable groups such as children and older adult (3, 4). Consequently, non-pharmacological approaches have gained growing attention in recent years as adjunctive strategies for managing pain and anxiety in emergency settings (5, 6).

Music, one of the most extensively studied non-pharmacological interventions, has shown therapeutic benefits across various clinical settings. Evidence from trials and reviews indicates that music can reduce preoperative anxiety, lessen postoperative pain, and enhance overall patient satisfaction (7, 8). The mechanisms underlying these effects are believed to include distraction from painful stimuli, modulation of autonomic nervous system activity, and emotional regulation through auditory stimulation (9, 10). Importantly, music is inexpensive, safe, and easy to implement without disrupting medical procedures. In emergency departments, where time constraints and high patient turnover are critical, music provides a practical, patient-centered adjunct to standard care (11, 12).

Despite growing evidence, the role of music interventions during emergency suturing remains insufficiently investigated. Some randomized controlled trials (RCTs) have reported that music listening during laceration repair reduces pain intensity and anxiety, whereas others have shown inconsistent results (13, 14). Variability in study design, music selection, intervention duration, and outcome measurement contributes to this uncertainty. Furthermore, the methodological quality of existing studies is inconsistent, with concerns related to blinding and small sample sizes (8, 15). Thus, a thorough meta-analysis of randomized controlled trials (RCTs) is needed to better define the effectiveness of music interventions in this setting. The present study sought to integrate the available evidence and evaluate how music influences pain and anxiety outcomes in patients receiving emergency suturing.

## 2 Methods

### 2.1 Literature search strategy

An extensive search of PubMed, Embase, Web of Science, and the Cochrane Library was performed in accordance with the PRISMA guidelines, covering publications up to September 9, 2025. The search strategy combined Medical Subject Headings (MeSH) with free-text terms, including “Emergency Service, Hospital,” “Wounds and Injuries,” and “Music.” Supplementary Table S1 provides the complete search strategy. In addition, two researchers (XZ and ZL) manually reviewed the reference lists of eligible studies and related reviews to identify further studies for inclusion. Data extraction and risk-of-bias assessment were also conducted independently by the same two reviewers, with any discrepancies resolved through discussion.

### 2.2 Inclusion and exclusion criteria

Studies were eligible if they satisfied the following criteria:

Population: Patients receiving laceration repair or suturing in an emergency department.

Intervention: Music provided during the suturing procedure.

Comparison: Standard care without music exposure.

Outcomes: Quantitative assessments of pain, anxiety, or other relevant clinical indicators.

Design: Randomized controlled trials (RCTs).

Exclusion criteria included: (1) conference abstracts, editorials, letters, case reports, reviews, or prior meta-analyses; (2) publications not in English; (3) animal studies; and (4) studies lacking outcome data relevant to the analysis.

### 2.3 Data extraction and quality assessment

Two reviewers (XZ and ZL) independently extracted data and assessed study quality, with discrepancies resolved through discussion or consultation with a third reviewer. Extracted information included year, country, patient age, sex ratio, sample size, laceration length (cm), number of sutures, procedure time (min), study design, and primary outcomes. The quality of the included randomized controlled trials was assessed with the Cochrane Risk of Bias Tool (16). This instrument examines seven domains: sequence generation, concealment of allocation, blinding of participants and staff, blinding of outcome assessment, completeness of outcome data, selective reporting, and other possible sources of bias.

### 2.4 Statistical methods

All statistical analyses were conducted with Review Manager (RevMan) version 5.3 (17) and Stata version 15. Standardized mean differences (SMDs) with 95% confidence intervals (CIs) were calculated for continuous variables, whereas dichotomous outcomes were analyzed using risk ratios (RRs) with 95% CIs. Heterogeneity was evaluated with the I^2^ statistic. When I^2^ was greater than 50% or substantial heterogeneity existed, a random-effects model was used; in other cases, a fixed-effects model was applied. Sensitivity analyses were conducted by removing individual studies one at a time to evaluate the stability of the results.

### 2.5 Publication bias

Egger’s test was applied to assess potential publication bias, with *p* < 0.05 considered evidence of significance.

### 2.6 GRADE rating

The certainty of the evidence was assessed with the Grading of Recommendations, Assessment, Development, and Evaluation (GRADE) framework (17). Based on this approach, evidence was categorized as high, moderate, low, or very low, considering factors such as risk of bias, inconsistency, indirectness, imprecision, and possible publication bias.

## 3 Results

### 3.1 Literature retrieval, study characteristics, and quality evaluation

**Figure 1:**
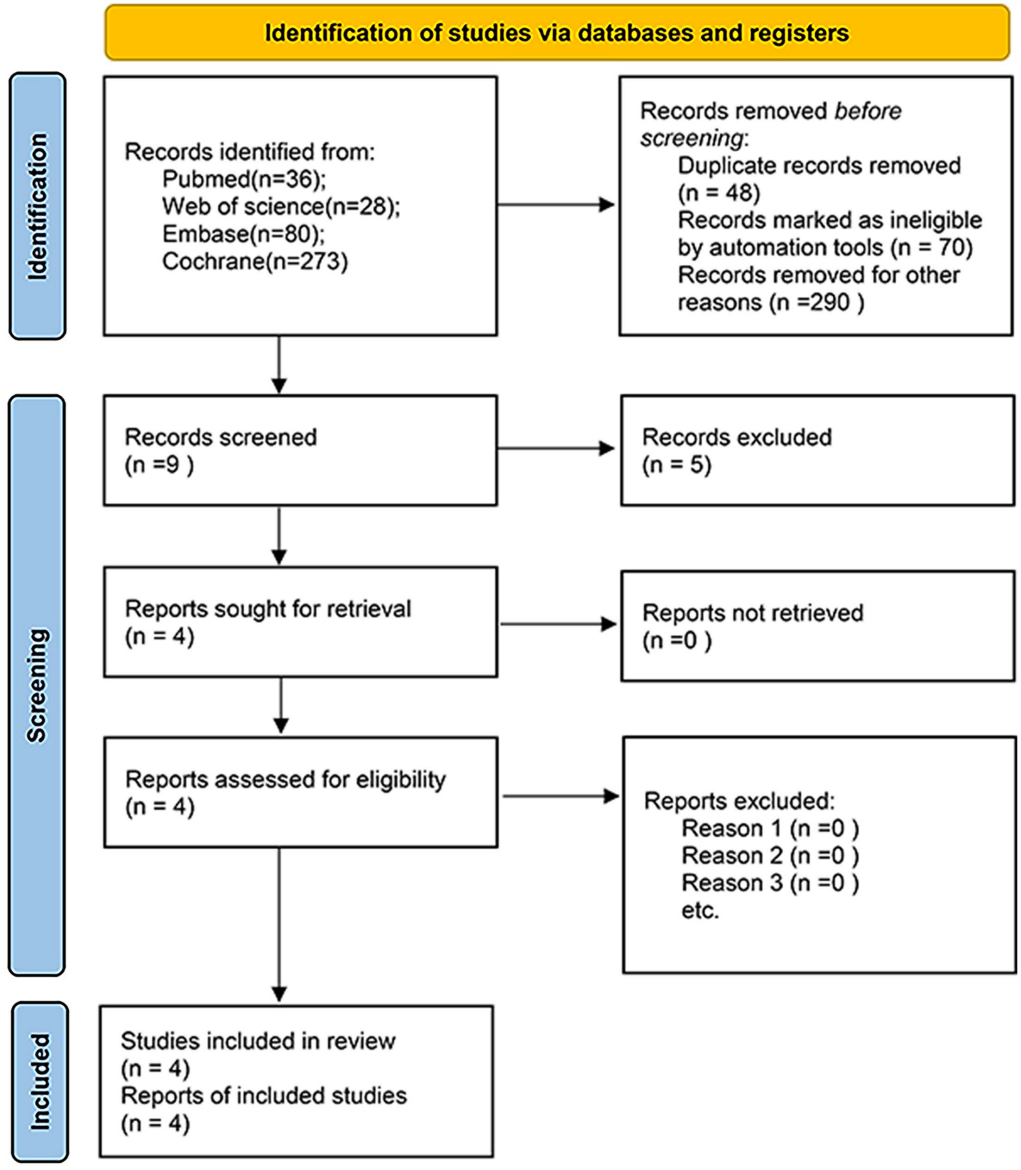
PRISMA flow diagram of study selection.

A systematic search of PubMed (*n =* 36), Embase (*n =* 80), Web of Science (*n =* 28), and the Cochrane Library (*n =* 273) identified 417 records. Following the removal of 48 duplicate records, 369 unique titles and abstracts were reviewed. Of these, 360 were excluded due to irrelevance, duplication, reviews or case reports, animal studies, or retracted publications. Nine full-text articles were reviewed for eligibility, and five were excluded due to insufficient data or other specified reasons. Ultimately, four RCTs (13, 18–20) were included in the meta-analysis (Figure 1).

In total, 388 patients were enrolled in the included trials, with 195 allocated to the music intervention group and 193 to the control group. The baseline characteristics of these studies are presented in Table 1.

**Table 1:** Basic characteristics of the literature.

Study	Year	Country	Age (year)	Male/Female	Sample size (*n*)	Laceration length (cm)	No. of sutures	Procedure time (min)	Study type	Outcomes
Menegazzi 1991	1991	Pittsburgh	Misic Group:24.4 ± 5.1 Control Group:25.9 ± 7.5	Misic Group:13/6 Control Group:8/11	Misic Group:19 Control Group:19	Misic Group:2.6 ± 1.3 Control Group:2.4 ± 1.3	Misic Group:6.4 ± 5.6 Control Group:5.3 ± 3.1	Misic Group:30.9 ± 30.8 Control Group:18.7 ± 9.1	RCTs	F1; F2
Hedayati 2023	2023	Iran	Misic Group:37.93 ± 12.61 Control Group:40.47 ± 15.35	Misic Group:17/13 Control Group:17/13	Misic Group:30 Control Group:30	Misic Group:3.653 ± 0.0917 Control Group:0.3733 ± 0.0976	Misic Group:3.83 ± 1.23 Control Group:3.77 ± 1.16	Misic Group:13.6 ± 3.93 Control Group:13.08 ± 4.63	RCTs	F1; F2
Bakhshandeh 2024	2024	Iran	Misic Group:4.83 ± 1.08 Control Group:4.70 ± 1.00	Misic Group:30/30 Control Group:30/30	Misic Group:60 Control Group:60	<3 cm	na	Misic Group:14.89 ± 0.323	RCTs	F1; F2
Charron 2025	2025	France	Misic Group:46.5 ± 18.8 Control Group:45.3 ± 18.2	Misic Group:65/19 Control Group:69/17	Misic Group:86 Control Group:84	na	na	na	RCTs	F1; F2

F1, Pain VAS; F2, Anxiety VAS; VAS, Visual Analogue Scale.

**Figure 2:**
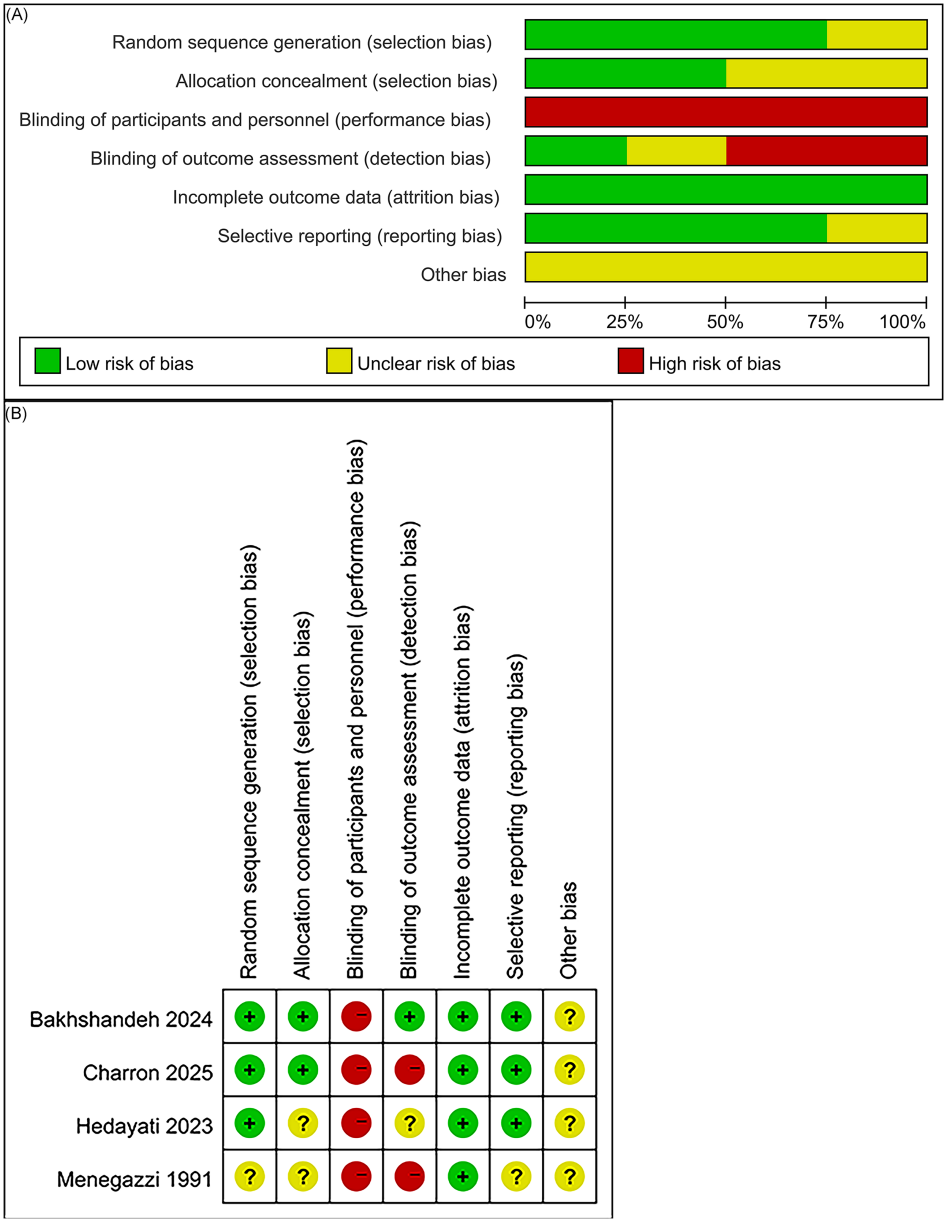
Risk of bias assessment of the included RCTs. **(A)** Summary of risk of bias; **(B)** Risk of bias judgments for individual studies.

The methodological quality of the trials was assessed using the Cochrane Risk of Bias Tool. As music intervention cannot feasibly be blinded, the risk of performance bias (blinding of participants and personnel) was generally high. In Bakhshandeh (18), pain scores were independently recorded by researchers and an emergency nurse, resulting in a low risk of detection bias (blinding of outcome assessment). In contrast, the remaining studies were rated as high risk for this domain. The overall risk of bias assessment is shown in Figures 2A,B.

### 3.2 Meta-analysis results

#### 3.2.1 Pain VAS

**Figure 3:**
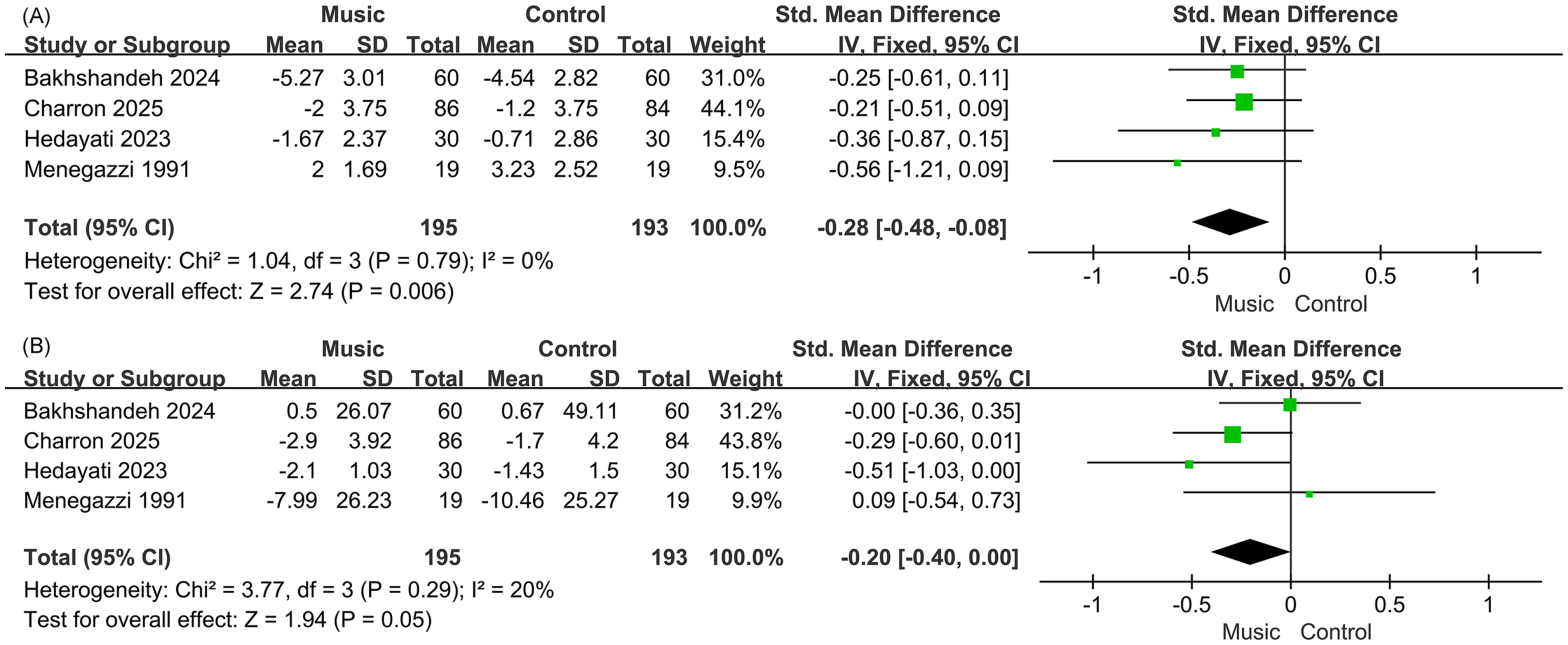
Forest plot **(A)**. Pain VAS; **(B)**. Anxiety VAS.

All four RCTs reported pain outcomes measured using the Visual Analogue Scale (VAS). The combined analysis indicated that music intervention led to a significant reduction in pain relative to the control group (SMD = −0.28, 95% CI: −0.48 to −0.08, *p* = 0.006). No statistical heterogeneity was detected (I^2^ = 0%, *p* = 0.79), indicating consistent results across studies (Figure 3A).

#### 3.2.2 Anxiety VAS

All four RCTs reported anxiety outcomes measured using the VAS. The pooled analysis indicated a trend toward reduced anxiety in the music group, although the effect was only marginally significant (SMD = −0.40, 95% CI: −0.80 to 0.00, *p* = 0.05). Low heterogeneity was detected (I^2^ = 20%, *p* = 0.29), suggesting acceptable consistency across studies (Figure 3B).

### 3.3 Publication bias and sensitivity analysis

**Figure 4:**
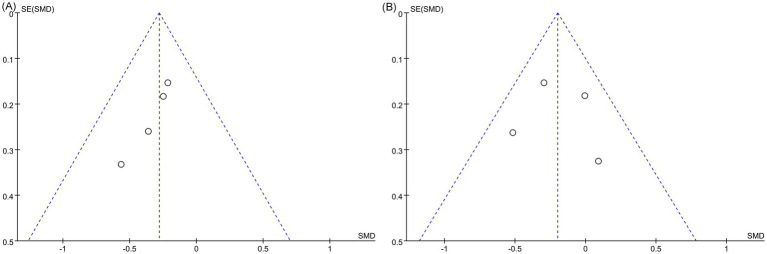
Funnel chart. **(A)**. Pain VAS; **(B)**. Anxiety VAS.

**Figure 5:**
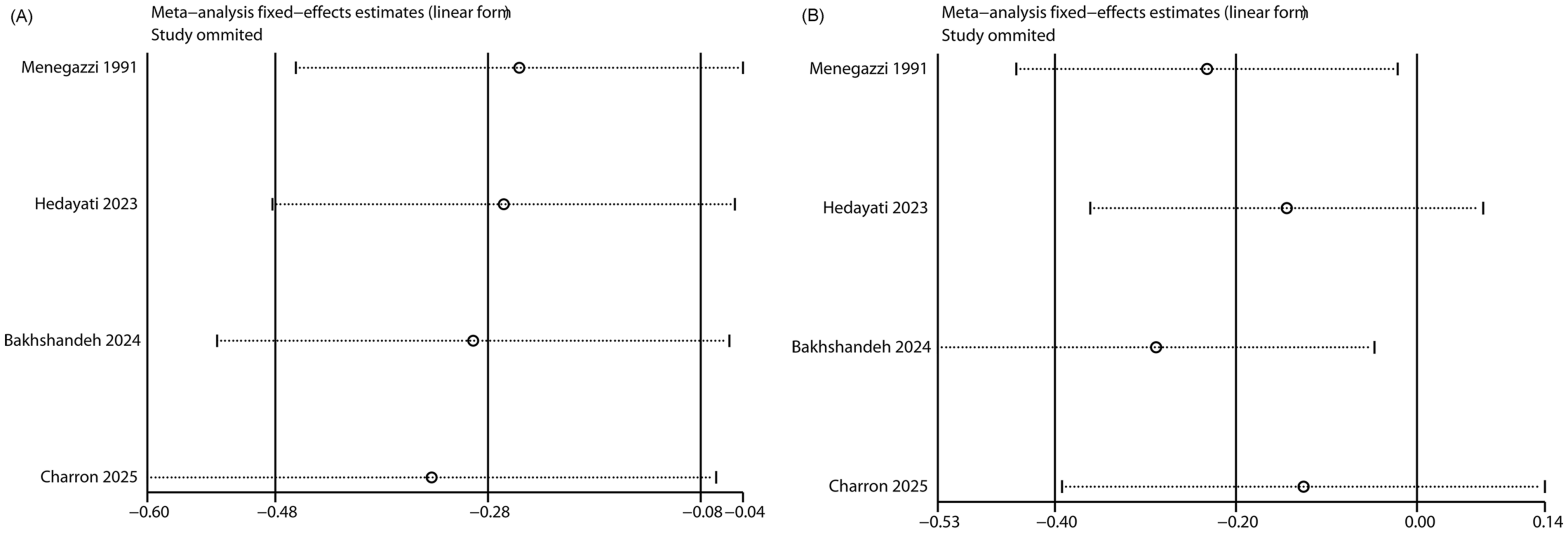
Sensitivity analysis. **(A)**. Pain VAS; **(B)**. Anxiety VAS.

Egger’s regression test was used to assess publication bias. No bias was observed for anxiety outcomes (*p* = 0.867), while evidence of bias was found for pain outcomes (*p* = 0.017). The funnel plot results were consistent with the Egger’s test results (Figures 4A,B). Sensitivity analyses were carried out by removing individual studies one at a time. The pooled results for both pain and anxiety outcomes remained consistent, reinforcing the robustness of the findings (Figures 5A,B).

### 3.4 GRADE quality of evidence assessment

According to the GRADE assessment, the certainty of evidence was low for Pain VAS and moderate for Anxiety VAS, mainly due to serious risk of bias. A detailed GRADE summary is presented in Table 2.

**Table 2:** GRADE classification quality of evidence.

Outcomes	Risk of bias	Inconsistency	Indirectness	Imprecision	Publication bias	Plausible confounding	Magnitude of effect	Dose–response gradient	Quality
Pain VAS	No serious	No serious	No serious	Serious	Serious	Would not reduce effect	No	No	Low
Anxiety VAS	No serious	No serious	No serious	Serious	No serious	Would not reduce effect	No	No	Moderate

## 4 Discussion

This meta-analysis provides evidence that music intervention during emergency suturing effectively reduces pain and may decrease anxiety in patients undergoing wound repair. By pooling data from four randomized controlled trials, music significantly reduced pain intensity (SMD = −0.28, 95% CI: −0.48 to −0.08) and demonstrated a borderline reduction in anxiety (SMD = −0.40, 95% CI: −0.80 to 0.00). Low heterogeneity across studies supported the consistency of findings. Sensitivity analyses confirmed robustness, while publication bias was detected for pain but not for anxiety outcomes. According to GRADE evaluation, the certainty of evidence ranged from low to moderate, primarily due to risks of bias related to blinding and small sample sizes. Collectively, these results suggest that music is a safe, inexpensive, and non-invasive adjunct to routine care during emergency suturing. The consistent reduction in pain, together with the trend toward anxiety relief, underscores the clinical potential of music therapy to improve patient experience in emergency departments. Although the anxiolytic effect did not reach clear statistical significance, the direction of effect was consistent with pain outcomes, suggesting a potential benefit that may have been underestimated due to contextual and methodological factors.

From a neurobiological perspective, music may reduce pain by modulating limbic system activity, including the amygdala, hippocampus, and anterior cingulate cortex, which are involved in emotional processing and the affective component of pain (21). By engaging attentional and emotional networks, music can divert cognitive resources from nociceptive stimuli and activate descending pain inhibitory pathways, thereby contributing to analgesic effects (22). In contrast, anxiety during emergency procedures may be more strongly driven by acute situational stressors, which could partly explain the more modest anxiolytic effects observed. Nevertheless, the consistent direction of effects across outcomes suggests that the benefits of music may extend beyond pain relief but be underestimated in emergency settings.

Our findings align with previous randomized studies examining the role of music during wound repair. Menegazzi et al. (20) first reported that music significantly reduced pain scores during laceration repair, although reductions in anxiety were not significant. More recent trials provide additional support: Bakhshandeh et al. (18) found that music reduced children’s pain during suturing and appeared to lower parental anxiety, although statistical significance was limited by small sample size. Similarly, Hedayati et al. (19) reported that music significantly reduced anxiety in adults undergoing wound repair and lowered pain without reaching statistical significance. In contrast, Charron et al. (13), in the EMERGENCE trial, observed no significant reduction in pain but did find significant improvements in anxiety as well as patient and physician satisfaction. Variation in outcomes across these trials may be explained by differences in music type, mode of delivery (live, recorded, or app-based), baseline pain levels, and outcome assessment. Importantly, anxiety outcomes may be particularly sensitive to differences in assessment timing and procedural context, which could contribute to the observed discordance between pain and anxiety effects across studies. Nonetheless, the overall direction of evidence supports the role of music in enhancing the procedural experience during emergency suturing.

Beyond suturing-specific studies, research in emergency and perioperative care supports these findings. Angkoontassaneeyarat et al. (23) reported that music therapy significantly reduced pain and anxiety in emergency department patients with non-trauma complaints, reinforcing its broader utility in acute care. Ortiz et al. (24) demonstrated additional benefits in pediatrics, where combining music therapy with child life interventions significantly reduced procedural distress during intravenous placement. These results align with earlier work by Tanabe et al. (25), who found that music improved satisfaction even when pain reduction was not clinically significant. Taken together, these studies support the notion that music can attenuate stress-related arousal, even when conventional anxiety scales fail to capture subtle psychological benefits in high-acuity settings. Collectively, these studies suggest that music functions as both a distractive and emotionally supportive intervention, with potential implications for reducing analgesic use and improving cooperation during invasive procedures.

The mechanisms underlying these effects are multifactorial. Music can function as a distractor, diverting attention from nociceptive input and reducing perceived pain intensity (26, 27). It also modulates autonomic activity, lowering heart rate, respiratory rate, and blood pressure, as demonstrated in the EMERGENCE trial (13). Neurophysiological studies suggest that music may stimulate endogenous opioid and dopamine release, contributing to analgesia and improved mood. Cultural and personal preferences are also important; Hedayati et al. (19) used traditional Iranian music, which may have enhanced receptivity in their population. Likewise, studies allowing patient-selected music, such as Menegazzi et al. (20), often reported greater reductions in pain. These findings highlight the importance of tailoring interventions—both in music selection and delivery method—to maximize clinical benefit.

However, in emergency departments, anxiety is often regarded as a physiological response to acute stress and is not routinely treated, whereas pain is systematically managed using local anesthetics. This imbalance may introduce a form of performance bias, whereby the standardized management of pain reduces variability and facilitates detection of adjunctive analgesic effects, while untreated anxiety remains more heterogeneous and susceptible to unmeasured psychological and environmental influences. Such context-specific bias may partly explain why the anxiolytic effect of music appeared less robust than its analgesic effect in this meta-analysis.

This study has several limitations. First, the number of included trials was small, with only four RCTs enrolling fewer than 400 patients in total, which reduced statistical power and limited generalizability. Second, blinding was inherently difficult, as patients were aware of receiving music, introducing performance bias. Third, intervention protocols varied considerably, with some studies using live or app-based music and others pre-recorded tracks, reducing direct comparability. Fourth, publication bias was detected for pain outcomes, raising the possibility that negative studies remain unpublished. Finally, although statistical heterogeneity was low, unmeasured factors such as cultural music preferences, procedural context, and baseline anxiety levels may have influenced outcomes. Future studies should incorporate standardized anxiety assessments, longer or pre-procedural exposure to music, and consideration of psychological support strategies to better delineate the role of music in mitigating anxiety during emergency procedures. Despite these limitations, the findings provide clinically meaningful insights and underscore the need for larger multicenter RCTs with standardized protocols to establish clear guidelines for integrating music interventions into emergency wound care.

## 5 Conclusion

This meta-analysis shows that music intervention during emergency suturing significantly reduces pain and demonstrates a trend toward decreasing anxiety, with consistent findings across randomized controlled trials. The differential magnitude of effect observed between pain and anxiety may reflect the specific emergency care context, in which pain is routinely managed with local anesthetics, whereas anxiety is often regarded as an expected physiological response to acute stress and is not systematically treated. As a non-invasive, low-cost, and easily implementable adjunct to standard emergency care, music represents a feasible strategy to enhance patient comfort and procedural tolerance during wound repair. Even when anxiolytic effects appear modest, music may contribute to attenuation of stress-related arousal and improvement in overall procedural tolerance. Although the certainty of evidence remains low to moderate due to methodological limitations, these findings support the practical integration of music intervention into routine emergency suturing. Future large, multicenter randomized controlled trials should incorporate standardized anxiety assessments and consider contextual sources of bias inherent to emergency settings, assess the influence of patient music preference, and develop clinical guidelines for integrating music into routine emergency wound care.

## Data Availability

The original contributions presented in the study are included in the article/Supplementary material, further inquiries can be directed to the corresponding author.
