# Supplementary material for: The impact of music intervention during emergency suturing on patients’ pain and anxiety: a meta-analysis
Source: Front Public Health. 2026 Jan 27;14:1713057. doi: 10.3389/fpubh.2026.1713057 (PMC12886440; doi:10.3389/fpubh.2026.1713057)
Supplement: Supplementary file 1 [file Data_Sheet_1.docx]

| Table S1 The searching strategy | | |
| --- | --- | --- |
| Databases | Searching strategy | Number of literature |
| Pubmed | ((((((((((((((((((((((((((((((((((((((((((((((((((((((Emergency Service, Hospital[MeSH Terms]) OR (Emergency Service, Hospital[Title/Abstract])) OR (Emergency Services, Hospital[Title/Abstract])) OR (Hospital Emergency Services[Title/Abstract])) OR (Services, Hospital Emergency[Title/Abstract])) OR (Hospital Emergency Service[Title/Abstract])) OR (Service, Hospital Emergency[Title/Abstract])) OR (Emergency Hospital Service[Title/Abstract])) OR (Emergency Hospital Services[Title/Abstract])) OR (Hospital Service, Emergency[Title/Abstract])) OR (Hospital Services, Emergency[Title/Abstract])) OR (Service, Emergency Hospital[Title/Abstract])) OR (Services, Emergency Hospital[Title/Abstract])) OR (hospital Service Emergency[Title/Abstract])) OR (Emergencies, Hospital Service[Title/Abstract])) OR (Emergency, Hospital Service[Title/Abstract])) OR (Hospital Service Emergencies[Title/Abstract])) OR (Service Emergencies, Hospital[Title/Abstract])) OR (Service Emergency, Hospital[Title/Abstract])) OR (Emergency Room[Title/Abstract])) OR (Emergency Rooms[Title/Abstract])) OR (Room, Emergency[Title/Abstract])) OR (Rooms, Emergency[Title/Abstract])) OR (Accident[Title/Abstract] AND Emergency Department[Title/Abstract])) OR (Emergency Ward[Title/Abstract])) OR (Emergency Wards[Title/Abstract])) OR (Ward, Emergency[Title/Abstract])) OR (Wards, Emergency[Title/Abstract])) OR (Emergency Departments[Title/Abstract])) OR (Department, Emergency[Title/Abstract])) OR (Departments, Emergency[Title/Abstract])) OR (Emergency Department[Title/Abstract])) OR (Emergency Units[Title/Abstract])) OR (Emergency Unit[Title/Abstract])) OR (Unit, Emergency[Title/Abstract])) OR (Units, Emergency[Title/Abstract])) OR (Hospital Emergency Room[Title/Abstract])) OR (Emergency Room, Hospital[Title/Abstract])) OR (Emergency Rooms, Hospital[Title/Abstract])) OR (Hospital Emergency Rooms[Title/Abstract])) OR (Room, Hospital Emergency[Title/Abstract])) OR (Rooms, Hospital Emergency[Title/Abstract])) OR (Emergency Outpatient Unit[Title/Abstract])) OR (Emergency Outpatient Units[Title/Abstract])) OR (Outpatient Unit, Emergency[Title/Abstract])) OR (Outpatient Units, Emergency[Title/Abstract])) OR (Unit, Emergency Outpatient[Title/Abstract])) OR (Units, Emergency Outpatient[Title/Abstract])) OR (Hospital Emergency Services Utilization[Title/Abstract])) OR (Emergency Services Utilization[Title/Abstract])) OR (Services Utilization, Emergency[Title/Abstract])) OR (Services Utilizations, Emergency[Title/Abstract])) OR (Utilization, Emergency Services[Title/Abstract])) AND (((((((((((((((((((((((((((Wounds and Injuries[MeSH Terms]) OR (Sutures[MeSH Terms])) OR (Wounds[Title/Abstract] AND Injuries[Title/Abstract])) OR (Injuries[Title/Abstract] AND Wounds[Title/Abstract])) OR (Injuries, Wounds[Title/Abstract])) OR (Wounds[Title/Abstract] AND Injury[Title/Abstract])) OR (Injury[Title/Abstract] AND Wounds[Title/Abstract])) OR (Wounds, Injury[Title/Abstract])) OR (Wounds[Title/Abstract])) OR (Wound[Title/Abstract])) OR (Injuries[Title/Abstract])) OR (Injury[Title/Abstract])) OR (Research-Related Injuries[Title/Abstract])) OR (Injury, Research-Related[Title/Abstract])) OR (Research Related Injuries[Title/Abstract])) OR (research-Related Injury[Title/Abstract])) OR (Physical Trauma[Title/Abstract])) OR (Physical Traumas[Title/Abstract])) OR (Trauma, Physical[Title/Abstract])) OR (Trauma[Title/Abstract])) OR (Traumas[Title/Abstract])) OR (Sutures[Title/Abstract])) OR (Suture[Title/Abstract])) OR (Staple, Surgical[Title/Abstract])) OR (Surgical Staple[Title/Abstract])) OR (Staples, Surgical[Title/Abstract])) OR (Surgical Staples[Title/Abstract]))) AND (((((((((((((((((Music[MeSH Terms]) OR (Music[Title/Abstract])) OR (Songs[Title/Abstract])) OR (Song[Title/Abstract])) OR (Vocal Melody[Title/Abstract])) OR (Melody, Vocal[Title/Abstract])) OR (Vocal Melodies[Title/Abstract])) OR (Classical Music[Title/Abstract])) OR (Music, Classical[Title/Abstract])) OR (Jazz Music[Title/Abstract])) OR (Music, Jazz[Title/Abstract])) OR (Rap Music[Title/Abstract])) OR (Music, Rap[Title/Abstract])) OR (Hip Hop Music[Title/Abstract])) OR (Hop Music, Hip[Title/Abstract])) OR (Music, Hip Hop[Title/Abstract])) OR (Rock[Title/Abstract] AND Roll Music[Title/Abstract])) | 36 |
| Web of science | 1 "TS=(Emergency Service, Hospital OR Emergency Services, Hospital OR Hospital Emergency Services OR Services, Hospital Emergency OR Hospital Emergency Service OR Service, Hospital Emergency OR Emergency Hospital Service OR Emergency Hospital Services OR Hospital Service, Emergency OR Hospital Services, Emergency OR Service, Emergency Hospital OR Services, Emergency Hospital OR Hospital Service Emergency OR Emergencies, Hospital Service OR Emergency, Hospital Service OR Hospital Service Emergencies OR Service Emergencies, Hospital OR Service Emergency, Hospital OR Emergency Room OR Emergency Rooms OR Room, Emergency OR Rooms, Emergency OR Accident and Emergency Department OR Emergency Ward OR Emergency Wards OR Ward, Emergency OR Wards, Emergency OR Emergency Departments OR Department, Emergency OR Departments, Emergency OR Emergency Department OR Emergency Units OR Emergency Unit OR Unit, Emergency OR Units, Emergency OR Hospital Emergency Room OR Emergency Room, Hospital OR Emergency Rooms, Hospital OR Hospital Emergency Rooms OR Room, Hospital Emergency OR Rooms, Hospital Emergency OR Emergency Outpatient Unit OR Emergency Outpatient Units OR Outpatient Unit, Emergency OR Outpatient Units, Emergency OR Unit, Emergency Outpatient OR Units, Emergency Outpatient OR Hospital Emergency Services Utilization OR Emergency Services Utilization OR Services Utilization, Emergency OR Services Utilizations, Emergency OR Utilization, Emergency Services) "  2 "TS=(Wounds and Injuries OR Injuries and Wounds OR Injuries, Wounds OR Wounds and Injury OR Injury and Wounds OR Wounds, Injury OR Wounds OR Wound OR Injuries OR Injury OR Research-Related Injuries OR Injury, Research-Related OR Research Related Injuries OR Research-Related Injury OR Physical Trauma OR Physical Traumas OR Trauma, Physical OR Trauma OR Traumas OR Sutures OR Suture OR Staple, Surgical OR Surgical Staple OR Staples, Surgical OR Surgical Staples) "  3 "TS=(Music OR Songs OR Song OR Vocal Melody OR Melodies, Vocal OR Melody, Vocal OR Vocal Melodies OR Classical Music OR Music, Classical OR Jazz Music OR Music, Jazz OR Rap Music OR Music, Rap OR Hip Hop Music OR Hop Music, Hip OR Music, Hip Hop OR Rock and Roll Music) "  4 "#3 AND #2 AND #1 " | 28 |
| Embase | #102 #54 AND #82 AND #101  #101 #83 OR #84 OR #85 OR #86 OR #87 OR #88 OR #89 OR #90 OR #91 OR #92 OR #93 OR #94 OR #95 OR #96 OR #97 OR #98 OR #99 OR #100  #100 'rock and roll music':ab,kw,ti  #99 'music, hip hop':ab,kw,ti  #98 'hop music, hip':ab,kw,ti  #97 'hip hop music':ab,kw,ti  #96 'music, rap':ab,kw,ti  #95 'rap music':ab,kw,ti  #94 'music, jazz':ab,kw,ti  #93 'jazz music':ab,kw,ti  #92 'music, classical':ab,kw,ti  #91 'classical music':ab,kw,ti  #90 'vocal melodies':ab,kw,ti  #89 'melody, vocal':ab,kw,ti  #88 'melodies, vocal':ab,kw,ti  #87 'vocal melody':ab,kw,ti  #86 'song':ab,kw,ti  #85 'songs':ab,kw,ti  #84 'music':ab,kw,ti  #83 'music'/exp  #82 #55 OR #56 OR #57 OR #58 OR #59 OR #60 OR #61 OR #62 OR #63 OR #64 OR #65 OR #66 OR #67 OR #68 OR #69 OR #70 OR #71 OR #72 OR #73 OR #74 OR #75 OR #76 OR #77 OR #78 OR #79 OR #80 OR #81  #81 'surgical staples':ab,kw,ti  #80 'staples, surgical':ab,kw,ti  #79 'surgical staple':ab,kw,ti  #78 'staple, surgical':ab,kw,ti  #77 'suture':ab,kw,ti  #76 'sutures':ab,kw,ti  #75 'sutures'/exp  #74 'traumas':ab,ti,kw  #73 'trauma':ab,ti,kw  #72 'trauma, physical':ab,ti,kw  #71 'physical traumas':ab,ti,kw  #70 'physical trauma':ab,ti,kw  #69 'research-related injury':ab,ti,kw  #68 'research related injuries':ab,ti,kw  #67 'injury, research-related':ab,ti,kw  #66 'research-related injuries':ab,ti,kw  #65 'injury':ab,ti,kw  #64 'injuries':ab,ti,kw  #63 'wound':ab,ti,kw  #62 'wounds':ab,ti,kw  #61 'wounds, injury':ab,ti,kw  #60 'injury and wounds':ab,ti,kw  #59 'wounds and injury':ab,ti,kw  #58 'injuries, wounds':ab,ti,kw  #57 'injuries and wounds':ab,ti,kw  #56 'wounds and injuries':ab,ti,kw  #55 'wounds and injuries'/exp  #54 #1 OR #2 OR #3 OR #4 OR #5 OR #6 OR #7 OR #8 OR #9 OR #10 OR #11 OR #12 OR #13 OR #14 OR #15 OR #16 OR #17 OR #18 OR #19 OR #20 OR #21 OR #22 OR #23 OR #24 OR #25 OR #26 OR #27 OR #28 OR #29 OR #30 OR #31 OR #32 OR #33 OR #34 OR #35 OR #36 OR #37 OR #38 OR #39 OR #40 OR #41 OR #42 OR #43 OR #44 OR #45 OR #46 OR #47 OR #48 OR #49 OR #50 OR #51 OR #52 OR #53  #53 'utilization, emergency services':ab,kw,ti  #52 'services utilizations, emergency':ab,kw,ti  #51 'services utilization, emergency':ab,kw,ti  #50 'emergency services utilization':ab,kw,ti  #49 'hospital emergency services utilization':ab,kw,ti  #48 'units, emergency outpatient':ab,kw,ti  #47 'unit, emergency outpatient':ab,kw,ti  #46 'outpatient units, emergency':ab,kw,ti  #45 'outpatient unit, emergency':ab,kw,ti  #44 'emergency outpatient units':ab,kw,ti  #43 'emergency outpatient unit':ab,kw,ti  #42 'rooms, hospital emergency':ab,kw,ti  #41 'room, hospital emergency':ab,kw,ti  #40 'hospital emergency rooms':ab,kw,ti  #39 'emergency rooms, hospital':ab,kw,ti  #38 'emergency room, hospital':ab,kw,ti  #37 'hospital emergency room':ab,kw,ti  #36 'units, emergency':ab,kw,ti  #35 'unit, emergency':ab,kw,ti  #34 'emergency unit':ab,kw,ti  #33 'emergency units':ab,kw,ti  #32 'emergency department':ab,kw,ti  #31 'departments, emergency':ab,kw,ti  #30 'department, emergency':ab,kw,ti  #29 'emergency departments':ab,kw,ti  #28 'wards, emergency':ab,kw,ti  #27 'ward, emergency':ab,kw,ti  #26 'emergency wards':ab,kw,ti  #25 'emergency ward':ab,kw,ti  #24 'accident and emergency department':ab,kw,ti  #23 'rooms, emergency':ab,kw,ti  #22 'room, emergency':ab,kw,ti  #21 'emergency rooms':ab,kw,ti  #20 'emergency room':ab,kw,ti  #19 'service emergency, hospital':ab,kw,ti  #18 'service emergencies, hospital':ab,kw,ti  #17 'hospital service emergencies':ab,kw,ti  #16 'emergency, hospital service':ab,kw,ti  #15 'emergencies, hospital service':ab,kw,ti  #14 'hospital service emergency':ab,kw,ti  #13 'services, emergency hospital':ab,kw,ti  #12 'service, emergency hospital':ab,kw,ti  #11 'hospital services, emergency':ab,kw,ti  #10 'hospital service, emergency':ab,kw,ti  #9 'emergency hospital services':ab,kw,ti  #8 'emergency hospital service':ab,kw,ti  #7 'service, hospital emergency':ab,kw,ti  #6 'hospital emergency service':ab,kw,ti  #5 'services, hospital emergency':ab,kw,ti  #4 'hospital emergency services':ab,kw,ti  #3 'emergency services, hospital':ab,kw,ti  #2 'emergency service, hospital':ab,kw,ti  #1 'emergency service, hospital'/exp | 80 |
| Cochrane | #1 (Emergency Service, Hospital):ti,ab,kw OR (Emergency Services, Hospital):ti,ab,kw OR (Hospital Emergency Services):ti,ab,kw OR (Services, Hospital Emergency):ti,ab,kw OR (Hospital Emergency Service):ti,ab,kw 7295  #2 (Service, Hospital Emergency):ti,ab,kw OR (Emergency Hospital Service):ti,ab,kw OR (Emergency Hospital Services):ti,ab,kw OR (Hospital Service, Emergency):ti,ab,kw OR (Hospital Services, Emergency):ti,ab,kw 7295  #3 (Service, Emergency Hospital):ti,ab,kw OR (Services, Emergency Hospital):ti,ab,kw OR (Hospital Service Emergency):ti,ab,kw OR (Emergencies, Hospital Service):ti,ab,kw OR (Emergency, Hospital Service):ti,ab,kw 7322  #4 (Hospital Service Emergencies):ti,ab,kw OR (Service Emergencies, Hospital):ti,ab,kw OR (Service Emergency, Hospital):ti,ab,kw OR (Emergency Room):ti,ab,kw OR (Emergency Rooms):ti,ab,kw 9550  #5 (Emergency Rooms):ti,ab,kw OR (Room, Emergency):ti,ab,kw OR (Rooms, Emergency):ti,ab,kw OR (Accident and Emergency Department):ti,ab,kw OR (Emergency Ward):ti,ab,kw 10463  #6 (Emergency Wards):ti,ab,kw OR (Ward, Emergency):ti,ab,kw OR (Wards, Emergency):ti,ab,kw OR (Emergency Departments):ti,ab,kw OR (Department, Emergency):ti,ab,kw 17926  #7 (Department, Emergency):ti,ab,kw OR (Departments, Emergency):ti,ab,kw OR (Emergency Department):ti,ab,kw OR (Emergency Units):ti,ab,kw OR (Emergency Unit):ti,ab,kw 18929  #8 (Emergency Unit):ti,ab,kw OR (Unit, Emergency):ti,ab,kw OR (Units, Emergency):ti,ab,kw OR (Hospital Emergency Room):ti,ab,kw OR (Emergency Room, Hospital):ti,ab,kw 6121  #9 (Emergency Rooms, Hospital):ti,ab,kw OR (Hospital Emergency Rooms):ti,ab,kw OR (Room, Hospital Emergency):ti,ab,kw OR (Rooms, Hospital Emergency):ti,ab,kw OR (Emergency Outpatient Unit):ti,ab,kw 2307  #10 (Emergency Outpatient Units):ti,ab,kw OR (Outpatient Unit, Emergency):ti,ab,kw OR (Outpatient Units, Emergency):ti,ab,kw OR (Unit, Emergency Outpatient):ti,ab,kw OR (Units, Emergency Outpatient):ti,ab,kw 336  #11 (Hospital Emergency Services Utilization):ti,ab,kw OR (Emergency Services Utilization):ti,ab,kw OR (Services Utilization, Emergency):ti,ab,kw OR (Services Utilizations, Emergency):ti,ab,kw OR (Utilization, Emergency Services):ti,ab,kw 844  #12 #1 OR #2 OR #3 OR #4 OR #5 OR #6 OR #7 OR #8 OR #9 OR #10 OR #11 24175  #13 (Wounds and Injuries):ti,ab,kw OR (Injuries and Wounds):ti,ab,kw OR (Injuries, Wounds):ti,ab,kw OR (Wounds and Injury):ti,ab,kw OR (Injury and Wounds):ti,ab,kw 6354  #14 (Wounds, Injury):ti,ab,kw OR (Wounds):ti,ab,kw OR (Wound):ti,ab,kw OR (Injuries):ti,ab,kw OR (Injury):ti,ab,kw 120598  #15 (Research-Related Injuries):ti,ab,kw OR (Injury, Research-Related):ti,ab,kw OR (Research Related Injuries):ti,ab,kw OR (Research-Related Injury):ti,ab,kw OR (Physical Trauma):ti,ab,kw 3826  #16 (Physical Traumas):ti,ab,kw OR (Trauma, Physical):ti,ab,kw OR (Trauma):ti,ab,kw OR (Traumas):ti,ab,kw OR (Sutures):ti,ab,kw 27491  #17 (Suture):ti,ab,kw OR (Staple, Surgical):ti,ab,kw OR (Surgical Staple):ti,ab,kw OR (Staples, Surgical):ti,ab,kw OR (Surgical Staples):ti,ab,kw 9474  #18 #13 OR #14 OR #15 OR #16 OR #17 139595  #19 (Music):ti,ab,kw OR (Songs):ti,ab,kw OR (Song):ti,ab,kw OR (Vocal Melody):ti,ab,kw OR (Melodies, Vocal):ti,ab,kw 8890  #20 (Melody, Vocal):ti,ab,kw OR (Vocal Melodies):ti,ab,kw OR (Classical Music):ti,ab,kw OR (Music, Classical):ti,ab,kw OR (Jazz Music):ti,ab,kw 452  #21 (Music, Jazz):ti,ab,kw OR (Rap Music):ti,ab,kw OR (Music, Rap):ti,ab,kw OR (Hip Hop Music):ti,ab,kw OR (Hop Music, Hip):ti,ab,kw 67  #22 (Music, Hip Hop):ti,ab,kw OR (Rock and Roll Music):ti,ab,kw 16  #23 #19 OR #20 OR #21 OR #22 8890  #24 #12 AND #15 AND #18 287 | 287 |
